# Patients return to sport after repair of anterior humeral avulsion of the glenohumeral ligament lesions: a systematic review

**DOI:** 10.1016/j.xrrt.2024.04.012

**Published:** 2024-05-09

**Authors:** Tyler C. Nicholson, Alexis B. Sandler, Lucas A. Georger, Kyle J. Klahs, John P. Scanaliato, Carolyn M. Hettrich, John C. Dunn, Nata Parnes

**Affiliations:** a Department of Orthopaedic Surgery and Rehabilitation, Texas Tech University Health Sciences Center, El Paso, TX, USA; b University of Missouri-Kansas City School of Medicine, Kansas City, MO, USA; c Midwest Ortho at RUSH University, Chicago, IL, USA; d Department of Orthopaedic Surgery, Carthage Area Hospital, Carthage, NY, USA; e Department of Orthopaedic Surgery, Claxton-Hepburn Medical Center, Ogdensburg, NY, USA

**Keywords:** Shoulder, Shoulder instability, HAGL, Humeral avulsion of the glenohumeral ligament, Return to sport, Return to play

## Abstract

**Background:**

Anterior humeral avulsions of the glenohumeral ligament (aHAGL) lesions are relatively rare causes of shoulder instability that affect athletes at a higher rate than other populations. The purpose of this study is to evaluate rate of return to sport (RTS) after HAGL repair.

**Methods:**

A search of the PubMed (MEDLINE), Scopus, and Cochrane CENTRAL databases was conducted on April 13, 2022 with the search terms “HAGL” or “humeral avulsion glenohumeral ligament” was used to conduct the systematic review. Inclusion criteria required that lesions were limited to aHAGL, axillary pouch or central HAGL, or both anterior and posterior HAGL lesions as specified by lesion description or direction of instability.

**Results:**

Screening and full-text manuscript review identified 7/967 studies eligible for inclusion with a total of 46 aHAGL lesions in athletes. Average rate of RTS was 93.5% (standard deviation [SD] = 13.4%, n = 43/46) with rate of RTS at previous levels of play averaging 80.0% (SD = 22.1%, n = 28/35). Neither rates of concomitant procedures nor concomitant pathology were associated with variation in RTS rates overall or level of RTS. Weighted average Rowe, subjective shoulder value, and Constant scores were 87.5 (SD = 4.9), 86.0 (SD = 2.0), and 82.2 (SD = 5.1), respectively, and 78.6% (n = 22/28) of patients reported postoperative satisfaction or “good/excellent” ratings following aHAGL repair. Adverse events occurred in 18.5% of patients (n = 10/54), most frequently recurrent instability (n = 3/54). Ultimately, 6.2% of patients eventually underwent reoperation (n = 3/17).

**Conclusion:**

As with other forms of anterior shoulder instability, RTS rates after aHAGL repair are high and many patients achieve their previous level of play. The most frequent adverse event was subjective recurrent instability with reoperation in 6.2% of patients. The findings from this study provide valuable pooled data on outcomes specific to aHAGL repair, particularly in the athlete population, and contribute to further understanding of outcomes regarding operative management of this rare pathology.

The inferior glenohumeral ligament is considered the most important anterior stabilizer of the glenohumeral joint and injury to this ligamentous complex that serves a critical role in maintaining shoulder stability is well described.^3^ Recurrent shoulder dislocation and subluxations are most commonly associated with glenoid labral tears. However, as early as 1942, Nicola^13^ first characterized an injury pattern caused by avulsion of the capsule from the anterior and inferior aspects of the humeral neck that he noted intraoperatively in a small series of patients presenting after acute dislocation. More than 4 decades later, this injury was termed a humeral avulsion of the glenohumeral ligament (HAGL) lesion and, despite their rarity, HAGL lesions have garnered substantial attention due to their increasingly recognized role in recurrent shoulder instability.^7^^,^^20^ The anterior band is implicated in 93% of inferior glenohumeral ligament injuries, rendering posterior band lesions considerably more rare.^19^

While the overall incidence of HAGL lesions is low, presentation often occurs concomitantly with other shoulder pathology and there is concern that HAGL lesions are underdiagnosed.^7^ Athletes, particularly those involved in contact sports, are especially predisposed to HAGL lesions. While Wolf et al^20^ reported a 9.3% incidence of HAGL lesions vs. a 73.5% incidence of Bankart lesions in patients with anterior shoulder instability, 72% of HAGL injuries have been linked to sports participation^2^ and among athletes with operatively managed shoulder instability, incidence of HAGL lesions was 25%.^14^

Successful nonoperative management of HAGL lesions has been reported;^5^ however, nonoperative management portends significantly higher recurrence rates, reported as high as 90%.^12^ Given the prohibitively high recurrence rates associated with nonoperative management, the majority of HAGL lesions are treated operatively, especially in the setting of concomitant injuries, recurrent dislocation, traumatic injuries, or injuries in young athletic patients.^11^ Subsequently, describing sports-related outcomes after surgical management of HAGL lesions is especially important in understanding this injury pattern. At this time, literature regarding HAGL lesions has primarily focused on its role in anterior shoulder instability and despite the demonstrated prevalence of this pathology within the athletic population, the overall impact of HAGL repair on return to sport (RTS) has not been adequately evaluated other than small case series.

The purpose of this study is to perform a systematic review to evaluate rate of RTS after HAGL repair. In assessing patient-reported outcomes, mean return time, and adverse events, we hypothesized that HAGL injury and ensuing operative HAGL repair facilitates an acceptable rate of RTS at a previous or higher level of play.

## Methods

### Eligibility

Preferred Reporting Items for Systematic Reviews and Meta-Analyses guidelines were referenced to conduct systematic review. Studies that reported rates of RTS among previously active patients who underwent arthroscopic or open HAGL repair with at least 1 year of follow-up on average were eligible for inclusion. HAGL lesions eligible for inclusion were limited to anterior HAGL (aHAGL), axillary pouch or central HAGL, or both anterior and posterior lesions as specified by lesion description or direction of instability. Exclusively posterior HAGL (pHAGL) lesions, bony aHAGL lesions, and reverse HAGL lesions were excluded, as were HAGL lesions undergoing revision procedures. In addition to RTS rates, studies reporting return to active-duty status within the military were also considered eligible for inclusion given the physical fitness standards requisite for these patients. Review articles, case reports with less than 3 patients, editorials, technical reports without clinical findings, cadaveric studies, biomechanical studies, and animal studies were not eligible for inclusion.

### Search

A search of the PubMed (MEDLINE), Scopus, and Cochrane CENTRAL databases was conducted on April 13, 2022 with the search terms “HAGL” or “humeral avulsion glenohumeral ligament”. No filters or limits were applied. Abstract screening, full-text review, and data extraction were performed by 3 authors (T.C.N., L.A.G., and A.B.S.).

### Data collection

Data related to patient demographics, athletic outcomes, functional outcomes, and adverse events were collected and pooled for analysis. Athletic activity–related data included the types of sports and number of participating athletes stratified into either competitive or contact/collision categories. Athletic outcomes were collected as rates of overall RTS in addition to rates stratified by RTS at previous or higher levels vs. RTS at a lower level. Patient-reported outcomes included pain as measured by the pain visual analog scale score; function as measured by the Rowe, Simple Shoulder Value, and Constant scores; and patient satisfaction rates. Adverse events included postoperative complications and reoperation.

### Statistics

Statistical significance was defined as α less than or equal to 0.05. Ranges of the rates of RTS were assessed and compared between studies. Pearson’s R was used to correlate concomitant shoulder pathology and rates of concomitant procedures in patients with HAGL with rates of RTS. Individual events and ranges of functional outcomes, rates of adverse events, and rates of reoperations were assessed and compared. Methodological Index for Non-Randomized Studies criteria were used to assess study quality, with noncomparative studies graded out of 16 and comparative studies out of 24.^18^

## Results

**Figure 1:**
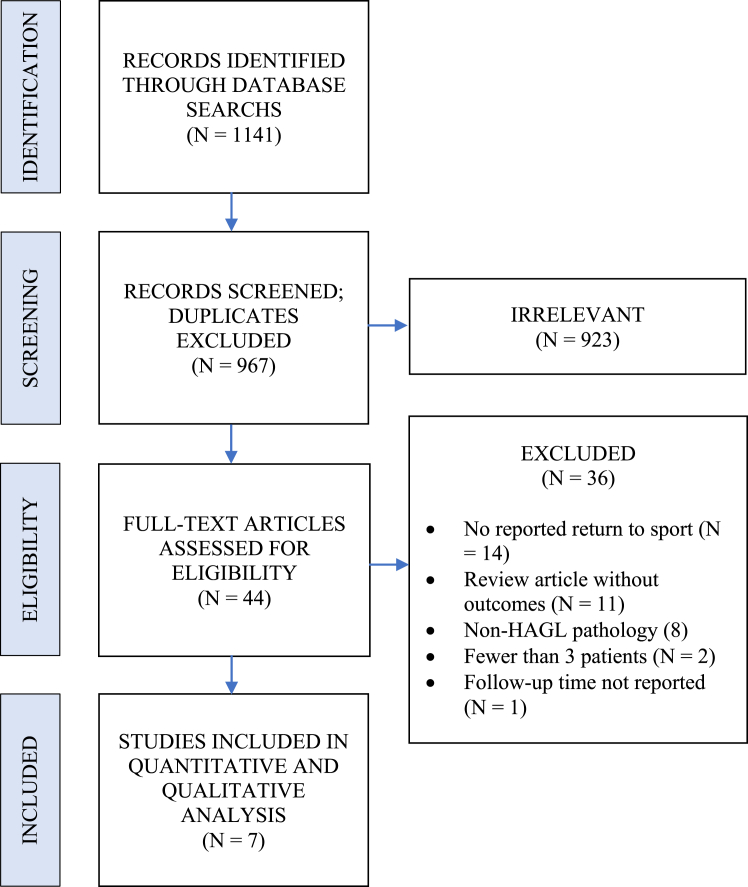
Inclusion flow diagram. *HAGL*, humeral avulsion of the glenohumeral ligament.

A total of 967 abstracts were screened, 44 manuscripts reviewed, and 7 studies were deemed eligible for inclusion (Fig. 1). Patient demographics and characteristics of included studies are presented in Table I. In total, 46 athletes of 59 patients were eligible for inclusion with an average follow-up of 48.9 months (standard deviation [SD] = 12.6). Most patients were male (60.4%, n = 32/53) with an average age of 23.7 years (SD = 3.4). The majority of patients underwent arthroscopic rather than open procedures (66.1%, n = 39/59). Inclusion criteria, exclusion criteria, concomitant injuries, and concomitant procedures are presented in Table II.

**Table I:** Demographics.

Study	Study design and level of evidence	HAGL lesions included	Total shoulders studied	Mean patient age (y)	Males (n)	Mean follow-up	MINORS score
Davey (2022)^4^	Cohort Study, III	15	15	21.5	15	53.5	24/24
Flury (2016)^6^	Case Series, IV	6	6	31	3	29	14/16
Kon (2005)^10^	Case Series, IV	3	3	25	2	19	14/16
Patzkowski (2019)^14^	Case Series, IV	9	36	20	0	59	13/16
Rhee (2007)^16^	Case Series, IV	6	6	28	NR	39	13/16
Schmiddem (2019)^17^	Case Series, IV	16	16	24	12	59	12/16
Taljanovic (2011)^19^	Case Series, IV	4	4	20	0	36	13/16

*HAGL*, humeral avulsion of the glenohumeral ligament; *MINORS*, Methodological Index for Non-Randomized Studies.

**Table II:** Inclusion and exclusion criteria.

Study	HAGL lesions included	Inclusion criteria	Exclusion criteria	Concomitant shoulder pathologies	Concomitant procedures	MINORS score
Davey (2022)^4^	15	Anterior shoulder instability with HAGL diagnosis by MRA that underwent open HAGL repair	Absence of MRA, follow-up less than 24 months	Labral Tear (14), HSL (10), RCT (3), Offtrack HSL (2), SLAP Lesion (2), Reverse HSL (1)	Labral repair (14)	24/24
Flury (2016)^6^	6	HAGL diagnosis with arthroscopic repair	None reported	Labral Tear (2), RCT (2)	RCR (2)	14/16
Kon (2005)^10^	3	Patients who underwent all-arthroscopic HAGL repair with suture anchors	None reported	HSL (2), Bankart Lesion (2)	Bankart repair (2)	14/16
Patzkowski (2019)^14^	9	Female patients at an NCAA Division I military service academy that underwent shoulder surgery with obtainable medical records	Male patients, revision surgical intervention for shoulder instability	Labral Tear (4), HSL (1)	NR	17/24
Rhee (2007)^16^	6	Patients with operatively managed anterior shoulder instability found to have HAGL lesions	None reported	Bankart Lesion (1), RCT (1), SLAP Lesion (1)	Bankart repair (1), SLAP repair (1)	13/16
Schmiddem (2019)^17^	16	Patients who underwent arthroscopic HAGL repair with lesions of at least 1.5 cm	None reported	Labral Tear (15), HSL (10), RCT (6), SLAP Lesion (2)	Labral repair (15), RCR (4), RCT débridement (2), SLAP repair (2)	12/16
Taljanovic (2011)^19^	4	Female college volleyball players with chronic, repetitive activity-related pain who were diagnosed with HAGL lesions on MRA	History of ipsilateral shoulder trauma, subluxation, or dislocation as well as sports other than volleyball	Labral tear (3), RCT (3)	Labral repair (3), RCT débridement (3)	13/16

*HAGL*, humeral avulsion of the glenohumeral ligament; *HSL*, Hill-Sachs Lesion; *MRA*, magnetic resonance angiography; *NR*, not reported; *RCR*, rotator cuff repair; *RCT*, rotator cuff tear; *SLAP*, superior labraum anterior to posterior; *NCAA*, National Collegiate Athletic Association; *MINORS*, Methodological Index for Non-Randomized Studies.

### Return to sport

In total, 46 athletes with aHAGL lesions were identified for inclusion. In the 6 studies specifying competitive and performance athletes, 93% (n = 40/43) of patients were considered competitive or performance athletes. Of the 6 studies specifying contact and collision sports, more than half of patients (59.5%, n = 22/37) were considered contact or collision athletes. Specific athletic activities are presented in Table III.

**Table III:** Athletic outcomes.

Study	Athletes with HAGL (n)	Competitive/Performance athletes with HAGL (n)	Contact/Collision athletes with HAGL (n)	Sports	RTS, % (n)	RTS at previous or higher level, % (n)
Davey (2022)^4^	14	14	14	Rugby (9), Gaelic Football (5)	100% (14)	86% (12)
Flury (2016)^6^	5	2	1	Football (1), Martial Arts (1)	100% (5)	100% (5)
Kon (2005)^10^	3	NR	1	Motocross (1), Baseball (1), Judo (1)	100% (3)	100% (3)
Patzkowski (2019)^14^	9	9	NR	Rugby (7), Military Obstacle Course (6), Boxing/Combat training (4), Basketball (4)∗	67% (6)	NR
Rhee (2007)^16^	2	2	1	Professional Hockey (1), Professional Volleyball (1)	100% (2)	NR
Schmiddem (2019)^17^	9	9	5	Australian Rules Football (5), Basketball (1), Rodeo (1)	100% (9)	44% (4)
Taljanovic (2011)^19^	4	4	0	Volleyball (4)	100% (4)	100% (4)

*HAGL*, humeral avulsion of the glenohumeral ligament; *NR*, not reported; *RTS*, return to sport.

∗ Data represent 36 students with shoulder pathology, 9 of which presented with HAGL.

**Figure 2:**
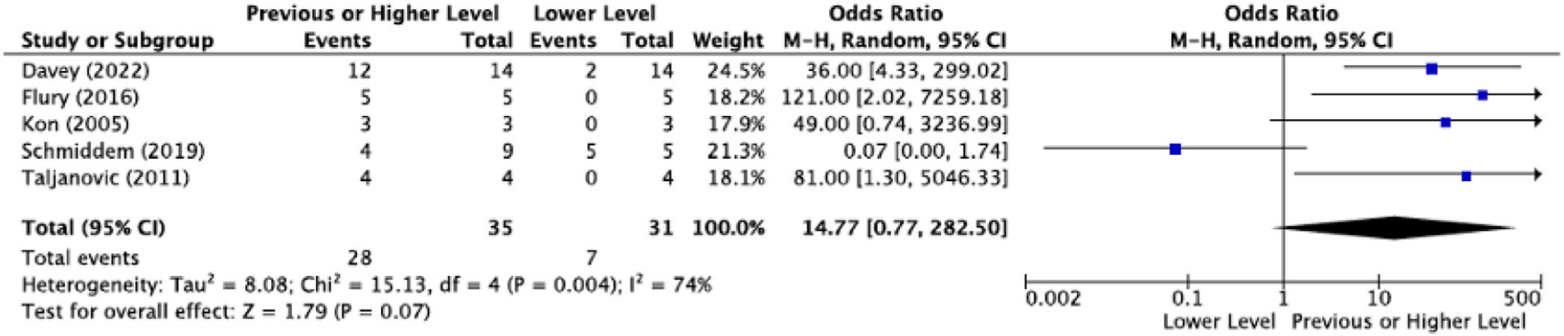
Forest plot of return to sport. *CI*, confidence interval; *M-H*, Mantel-Haenszel.

Average rate of RTS after HAGL repair was 93.5% (SD = 13.4%, range = 66%-100%; n = 43/46) with rate of RTS at previous levels of play averaging 80.0% (SD = 22.1%, range = 44%-100%; n = 28/35). The odds of return to play at previous level as compared to RTS at lower level vary significantly between studies (I^2^ = 74%, *P* = .004) with an odds ratio of 14.8 (confidence interval = 0.77-282.5, Z = 1.79, *P* = .07), with all studies except Schmiddem et al^17^ reporting higher rates of RTS at patients’ previous functional status (Fig. 2). The mean average time of return to play was 5.7 months (SD = 0.67, n = 18). As expected, there were significantly higher rates of concomitant procedures performed in patients with concomitant pathology (r(5) = 0.825, *P* = .022); however, neither rates of concomitant procedures or concomitant pathology were associated with variation in RTS rates (r(5) = 0.570, *P* = .182 and r(5) = 0.441, *P* = .322, respectively). Similarly, rates of RTS at previous or higher level did not correlate with rates of concomitant procedures or pathology (r(3) = 0.368, *P* = .543 and r(3) = 0.51, *P* = .935, respectively).

### Patient-reported outcomes

Only 1 study^19^ reported pain visual analog scale with a mean of 1.6 (SD = 2.6, n = 15). Weighted average Rowe score was 87.5 (SD = 4.9, n = 46), Simple Shoulder Value was 86.0 (SD = 2.0, n = 21), and Constant score, which includes both patient-reported and objective measures, was 82.2 (SD = 5.1, n = 12). A majority (78.6%, n = 22/28, range = 86.7%-100%) of patients reported either satisfaction or “good/excellent” ratings following aHAGL repair.

### Adverse events

Among all patients included in the 5 studies that reported adverse events, complications were noted in 18.5% of patients (n = 10/54, range = 0%-46.7%) (Table IV). Of these, subjective instability was the most common (n = 8/10, range = 11.1%-46.7%) with 4 patients reporting apprehension, 3 reporting recurrent instability, and 1 reporting subluxation. Four studies reported reoperation rates^1^^,^^4^^,^^8^^,^^19^ and 6.2% of patients ultimately underwent reoperation (n = 3/17, range = 0%-13.3%). Reoperations included revision with open Latarjet (n = 1), arthroscopic revision HAGL repair (n = 1), and biceps tenodesis (n = 1).

**Table IV:** Adverse events.

	Adverse events, % (n)	Adverse events description	Reoperations, % (n)	Reoperation procedures
Davey (2022)^4^	47% (7)	Apprehension (4), Subluxation (1), Recurrent instability (2)	13% (2)	Revision with open Latarjet (1), biceps tenodesis (1)
Flury (2016)^6^	33% (2)	AEs unrelated to shoulder, unspecified (2)	0% (0)	0
Patzkowski (2019)^14^	11% (1)	Recurrent instability (1)	11% (1)	Arthroscopic revision (1)
Rhee (2007)^16^	0% (0)	0	0% (0)	0
Schmiddem (2019)^17^	0% (0)	0	0% (0)	0

## Discussion

The purpose of the study was to determine the rate of RTS among athletes following surgical repair of aHAGL lesions. There was an overall high rate of RTS with the distinction of RTS at a level equal to preinjury play reported at 44.4% in one study,^4^ 85.7% in another,^6^ and 100% in the remaining 3.^4^^,^^10^^,^^19^

A number of recent studies have evaluated similar outcomes after HAGL repair with mixed results. Schmidden et al^17^ evaluated 18 arthroscopic HAGL repairs and reported follow-up among only 9 athletes. With the already limited followed patients, only 4 (44%) were able to return to the same level of play. The authors surmised that such low rates of RTS at prior level of play were linked to concomitant injuries that may have impacted RTS, a conclusion that was not universally found across the other studies in our systematic review. Additionally, this evaluation demonstrates that concomitant pathology and related procedures are a norm in the evaluation and management of HAGL injuries, suggesting that combined shoulder injuries and procedures are expected. Another recent study of 23 patients with aHAGL and pHAGL lesions found that while the majority of patients’ symptoms resolved at 20.2 months, only 6 of 12 athletes (50%) were able to return to some level of sport.^8^ The authors conjecture that low rates of return to preinjury level of performance may be attributed to failure to heal repaired HAGL lesions or concomitant injuries, prompting them to consider alternative means of HAGL repair. Our evaluation did not corroborate these relatively low levels of RTS and instead described substantially higher levels of RTS, even at previous or higher levels of play.

Other recent studies have noted a much higher rate of RTS than previously reported. In a retrospective review of matched groups with 24 months of follow-up, Davey et al^4^ reported RTS at 93.3% with 80% returning to their preinjury level of performance following open HAGL repair. These findings were not significantly different as compared to the rate found in the comparison group of patients experiencing non-HAGL–related anterior shoulder instability.^4^ In their 2017 systematic review of 18 studies evaluating indications for operative intervention in HAGL lesions, Bozzo et al^2^ similarly note that only 2 of 79 patients were unable to RTS, generating an overall RTS rate of 97%. The results of the present review demonstrate favorable RTS rates, which we believe offers a more accurate and updated evaluation of RTS following operative treatment of this relatively rare injury pattern.

The rates of RTS observed among studies included in our evaluation are comparable to published works evaluating RTS in patients experiencing anterior shoulder instability for reasons other than HAGL lesions. In a systematic review and meta-analysis evaluating RTS following anterior shoulder stabilization, Ialenti et al^9^ reported a same-level RTS rate ranging from 66% in patients undergoing open repair to 73% in patients undergoing Latarjet procedures with rates of RTS at any level ranging from 89% to 91%, respectively. Similarly, a systematic review of 16 articles by Abdul-Rassoul et al^1^ demonstrated an RTS rate ranging from 97.5% after arthroscopic Bankart repair to 83.6% after open Latarjet with rates of RTS at preinjury levels ranging from 92% to 69%. In the present study, our results demonstrate similarly high rates of RTS despite slightly lower rates of return to preinjury level of play. Ultimately, patients undergoing HAGL repair can expect a similar level of RTS and return to preinjury level of play as patients undergoing other stabilization procedures for anterior shoulder instability.

Of the 7 included studies, only 5 adequately reported complications. The overall pooled rate of adverse events was found to be 18.5%, with just under a third (30%) of patients with adverse events undergoing reoperation. In their study of aHAGL and pHAGL lesions, Grundshtein et al^8^ note complications in 6 of 23 patients (26%), with the most common complication related to anchor failure stemming from poor insertion technique. Additionally, 3 patients in their series required reoperation for instability or unrecognized concomitant injury. Davey et al^4^ reported a similarly high rate of postoperative complications following open HAGL repair, which occurred in 7 of 15 patients (47%). These complication rates were primarily related to subjective or objective instability, with 4 patients experiencing apprehension, 1 with subluxation, and 2 with recurrent instability. Revision surgery was required in 1 patient who was revised to open Latarjet, while 1 additional patient required reoperation for ongoing biceps pathology and eventual biceps tenodesis. Although they did not study rates of RTS, Provencher et al^15^ reported no observed serious postoperative complications at a minimum of 2-year postoperative follow-up in their study on HAGL repair. Ultimately, there is significant variability and heterogeneity observed among complications following HAGL repair in existing literature.

### Limitations

This study is not without limitations. Being a systematic review, this evaluation is limited by both the quantity and the quality of available data, which currently consists of a very limited number of studies typically classified as level 3 or 4 evidence. This illustrates the need for higher level studies evaluating HAGL lesions, treatments, and outcomes. Additionally, variability among sports participation, both contact and noncontact, contributes to heterogeneity among understanding RTS rates. There is also substantial variability in the methods used for reporting HAGL lesions, their location, concomitant shoulder pathology/procedures, techniques used for repair, and outcomes following treatment. We did not investigate reoperation due to an unrecognized HAGL, which would be an area for future study.

## Conclusion

As with other forms of anterior shoulder instability, RTS rates after aHAGL repair are high and many patients achieve their previous level of play. Adverse events are not uncommon, with subjective recurrent instability the most frequently reported. Patients and surgeons should also be aware that reoperation is not an insignificant risk. The findings from this study provide valuable pooled data on outcomes specific to aHAGL repair, particularly in the athlete population, and contribute to further understanding of outcomes regarding operative management of this rare pathology.

## Disclaimers:

Funding: This research did not receive any specific grant from funding agencies in the public, commercial, or not-for-profit sectors.

Conflicts of interest: Each author certifies that they have no commercial associations (eg, consultancies, stock ownership, equity interest, patent/licensing arrangements, etc.) that might pose a conflict of interest in connection with the submitted article.
